# Supplementary figures and images for: Microbial growth in biobeds for treatment of residual pesticide in banana plantations
Source: PeerJ. 2021 Sep 22;9:e12200. doi: 10.7717/peerj.12200 (PMC8464193; doi:10.7717/peerj.12200)

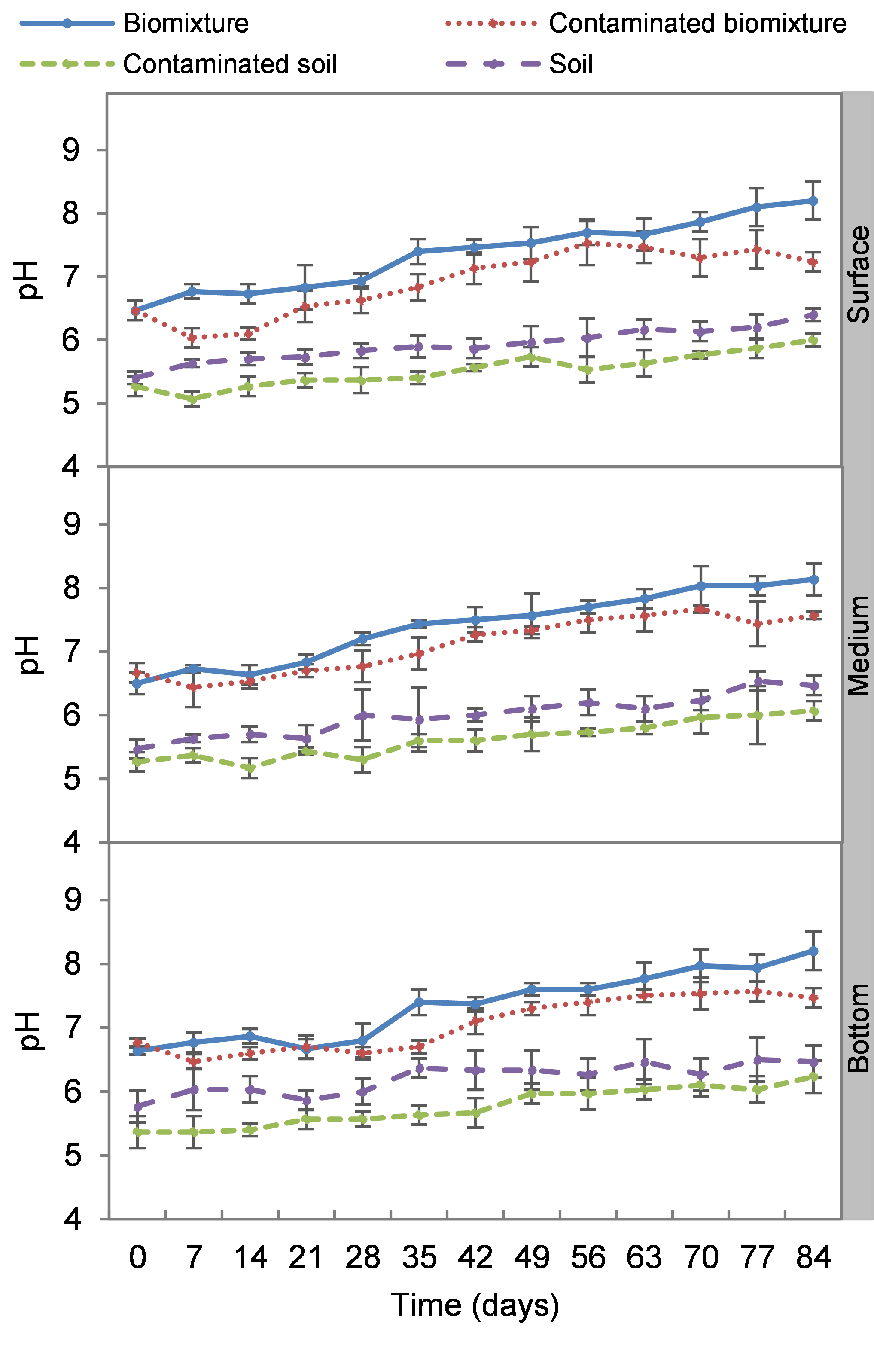

Supplement: Supplemental Information 2 — Error bars represent the standard deviation of three replications. [file peerj-09-12200-s002.png]

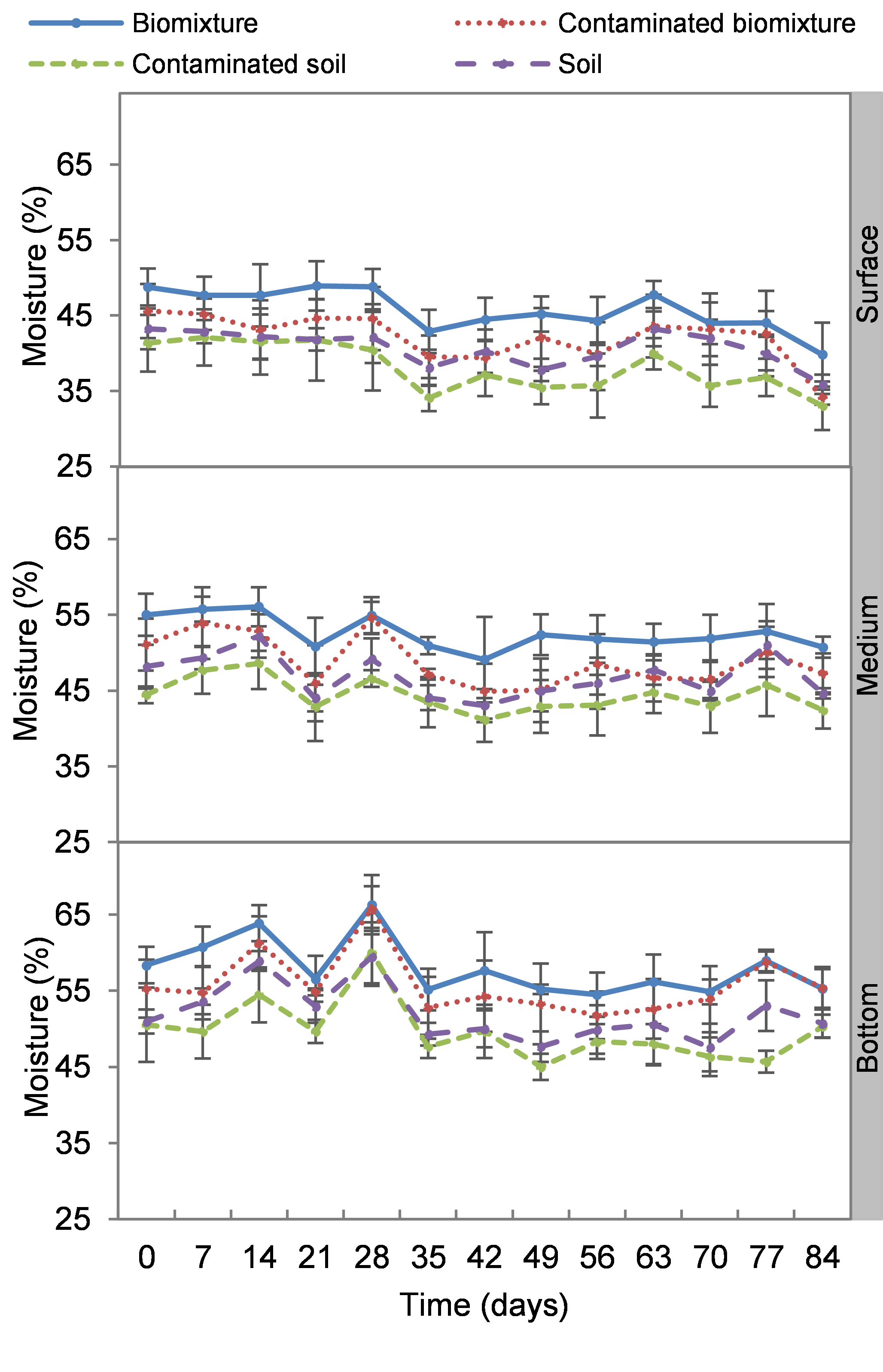

Supplement: Supplemental Information 3 — Error bars represent the standard deviation of three replications. [file peerj-09-12200-s003.png]

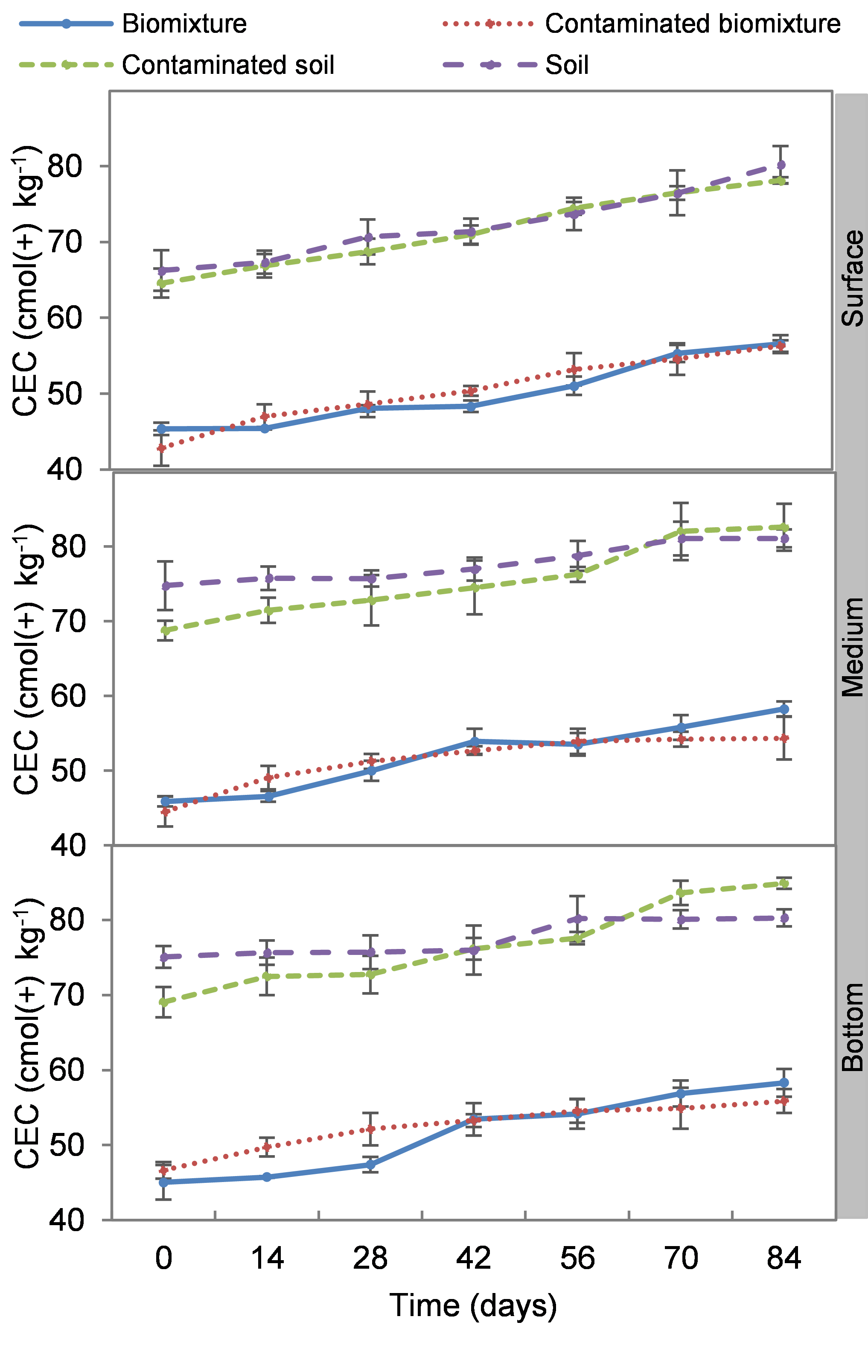

Supplement: Supplemental Information 4 — Error bars represent the standard deviation of three replications. [file peerj-09-12200-s004.png]

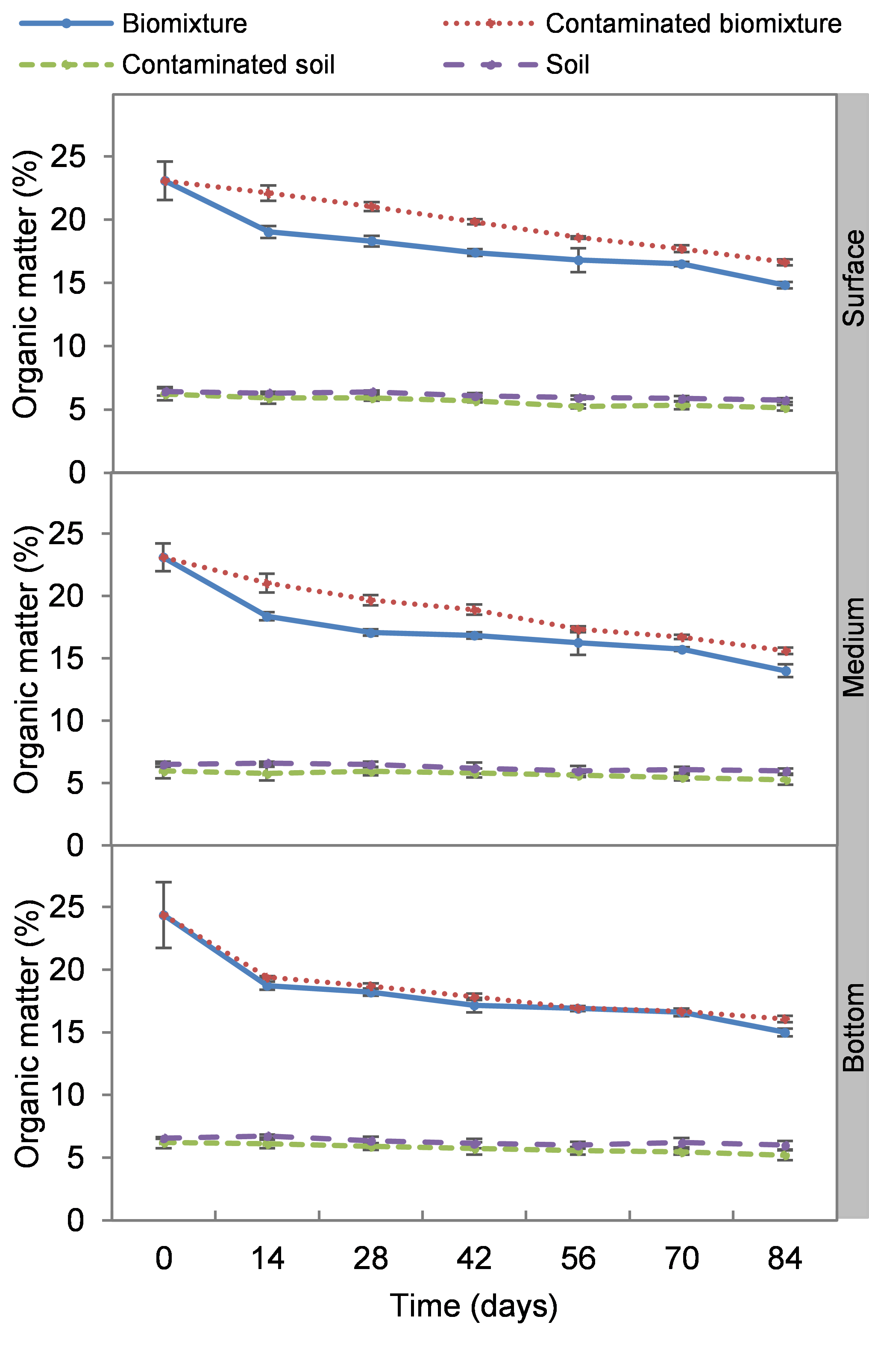

Supplement: Supplemental Information 5 — Error bars represent the standard deviation of three replications. [file peerj-09-12200-s005.png]
